# Gene Expression Network Reconstruction by Convex Feature Selection when Incorporating Genetic Perturbations

**DOI:** 10.1371/journal.pcbi.1001014

**Published:** 2010-12-02

**Authors:** Benjamin A. Logsdon, Jason Mezey

**Affiliations:** 1 Department of Biological Statistics and Computational Biology, Cornell University, Ithaca, New York, United States of America; 2 Department of Genetic Medicine, Weill Cornell Medical College, New York, New York, United States of America; University of Wisconsin-Madison, United States of America

## Abstract

Cellular gene expression measurements contain regulatory information that can be used to discover novel network relationships. Here, we present a new algorithm for network reconstruction powered by the adaptive lasso, a theoretically and empirically well-behaved method for selecting the regulatory features of a network. Any algorithms designed for network discovery that make use of directed probabilistic graphs require perturbations, produced by either experiments or naturally occurring genetic variation, to successfully infer unique regulatory relationships from gene expression data. Our approach makes use of appropriately selected *cis*-expression Quantitative Trait Loci (*cis*-eQTL), which provide a sufficient set of independent perturbations for maximum network resolution. We compare the performance of our network reconstruction algorithm to four other approaches: the PC-algorithm, QTLnet, the QDG algorithm, and the NEO algorithm, all of which have been used to reconstruct directed networks among phenotypes leveraging QTL. We show that the adaptive lasso can outperform these algorithms for networks of ten genes and ten *cis*-eQTL, and is competitive with the QDG algorithm for networks with thirty genes and thirty *cis*-eQTL, with rich topologies and hundreds of samples. Using this novel approach, we identify unique sets of directed relationships in *Saccharomyces cerevisiae* when analyzing genome-wide gene expression data for an intercross between a wild strain and a lab strain. We recover novel putative network relationships between a tyrosine biosynthesis gene (TYR1), and genes involved in endocytosis (RCY1), the spindle checkpoint (BUB2), sulfonate catabolism (JLP1), and cell-cell communication (PRM7). Our algorithm provides a synthesis of feature selection methods and graphical model theory that has the potential to reveal new directed regulatory relationships from the analysis of population level genetic and gene expression data.

## Author Summary

Determining a unique set of regulatory relationships underlying the observed expression of genes is a challenging problem, not only because of the many possible regulatory relationships, but also because highly distinct regulatory relationships can fit data equally well. In addition, most expression data-sets have relatively small sample sizes compared to the number of genes measured, causing high sampling variability that leads to a significant reduction in power and inflation of the false positive rate for any network reconstruction method. We propose a novel algorithm for network reconstruction that uses a theoretically and empirically well-behaved method for selecting regulatory features, while leveraging genetic perturbations arising from *cis*-expression Quantitative Trait Loci (*cis*-eQTL) to maximally resolve a network. Our algorithm has good performance for realistic samples sizes and can be used to identify a unique set of acyclic or cyclic regulatory relationships that explain observed gene expression.

## Introduction

Network analyses are increasingly applied to genome-wide gene expression data to infer regulatory relationships among genes and to understand the basis of complex disease [1], [2]. Probabilistic graphical techniques, which model genes as nodes and the conditional dependencies among genes as edges, are among the most frequently applied methods for this purpose. A diversity of such approaches have been proposed including Bayesian networks [3]–[5], undirected networks [6]–[8], and directed cyclic networks [9]–[11]. The popularity of these methods derives, in part, from the structure of these models that is well suited to algorithm development and because the network representation of these models can be used to construct specific biological hypotheses about the processes governing the activity of genes in a system [3]. As an example of this latter property, genes connected by an edge may indicate (at least) one of the genes is regulated by the other.

In graphical network inference, a theoretical principle that is now well appreciated [5], [10]–[17] is that ‘perturbations’ of the network can be leveraged to reduce the set of possible networks that can equivalently explain gene expression. In fact, since equivalent models can indicate conflicting regulatory relationships, perturbations are often necessary to extract regulatory relationships with any confidence. If the perturbations are controlled (e.g. knockouts of single genes), then a network among *n* genes can be recovered very efficiently with *n* knockouts [12]. Alternatively, perturbations that arise from naturally segregating variants, or combinations of genetic variants produced from carefully designed crosses, can also be leveraged [5], [10], [11], [13]–[19]. Perturbations of this type, caused by genetic polymorphisms in a population that alter the expression of genes across a population sample, are expression quantitative trait loci (eQTL) [15].

Despite the acknowledged importance of perturbations in network analysis, there has been little theoretical work concerning sets of perturbations that maximally limit the set of equivalent models for arbitrary directed networks. Limiting the set of equivalent models is of particular concern in cases where the true network has cyclic structure, where the set of statistically indistinguishable models may include drastically different topologies [20]. In this paper, we present theory concerning a minimally sufficient set of (genetic) perturbations to infer a maximally limited equivalent set of network architectures, which can subsequently be reconstructed using a single, convex optimization procedure. We demonstrate that for a specific type of network among both genes expression products and genotypes (an interaction or conditional independence network [21]), when including an appropriate set of genetic perturbations for the genotypes, specifically locally occurring *cis*-eQTL [14], the interaction network contains all the information necessary for directed network reconstruction. We can therefore estimate the regulatory relationships or features of a network directly from the interaction network with many different approaches [7], [8], [22]–[24]. Here, we use the adaptive lasso [25], a convex optimization procedure, to efficiently solve this model selection problem. This approach allows us to avoid the reliance on computationally inefficient heuristics [3]–[5], [10], [11], [16], [18], [19] with non-unique solutions, which can generate many possibly poor-fitting networks when considering sample sizes that are typical of experiments collecting genome-wide gene expression data.

Our algorithm includes three steps. First, an association analysis is carried out to identify strong local (*cis*-eQTL) perturbations of gene expression. Second, we combine the gene expression data and genotypes for the *cis*-eQTL, and use an adaptive lasso regression procedure [8], [25] to identify an interaction network [21] among gene expression products and *cis*-eQTL genotypes. The novel component of our algorithm is incorporated into this step, where we can immediately extract a unique, directed acyclic or cyclic network, given each gene in the network analysis has a unique *cis*-eQTL. Third, to ensure the edges in the interaction network correspond to the correct dependencies in the directed graph, we do a permutation test to ensure marginal independence between the *cis*-eQTL and the upstream gene based on the undirected edges recovered. We only use genetic perturbations that are *cis*-eQTL because of empirical evidence that local genetic polymorphism tends to have larger effects than *trans*-eQTL [26]–[28], and are therefore statistically more likely to be linked to locally causal variants. If the true network is a directed cyclic graph and if one uses *trans*-eQTL to attempt to find the true model, there can still be a larger equivalence class of models, since there is no way to know which gene a *trans*-eQTL actually feeds into in a cyclic graph because of equivalence (this is shown in the “Recovery” Theorem in the Methods). Our approach mirrors directed network inference approaches that seek to identify conditional independence and dependence relationships but avoids a computationally demanding step of iteratively testing for these relationships [11], [20], [29], [30].

To test this algorithm, we explore performance for simulated data. Specifically, the simulations are designed to capture scenarios where the underlying network is relatively sparse, and the strength of both the *cis*-eQTL and regulatory relationships is strong enough to detect given a relatively small numbers of samples, on the order of the number of genes being tested. We investigated networks of modest size (either 10 or 30 genes), since we wished to focus on cases where the set of genes being tested have strong *cis*-eQTL in linkage equilibrium, which in a typical eQTL genome-wide association study will be much smaller than the total number of genes being tested, [27], [28]. As a benchmark, we compare the performance of our algorithm to the PC-algorithm [29], [31], the QDG algorithm [14], the QTLnet algorithm [16], and the NEO algorithm [18]. We find that our algorithm can outperform all of these approaches in terms of controlling the false-discovery rate, and having greater power (given a large enough sample size) for the recovery of directed acyclic graphs and directed cyclic graphs. To empirically assess our algorithm, we also analyze data from a well powered intercross study in yeast [27]. From this analysis, we identify 35 genes with strong, independent *cis*-eQTL, and leveraged these perturbations to identify novel interactions. While we analyze the data from an intercross, both the theoretical results as well as the algorithm itself can be applied to natural populations as well.

## Results

### The gene expression network model

**Figure 1:**
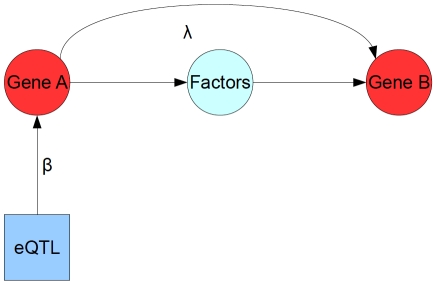
Example of biological relationships that can be reconstructed by the algorithm. An expression Quantitative Trait Locus (eQTL) directly alters the expression level of Gene A, a relationship that we represent in our network model with the parameter 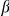. This gene in turn has an effect on Gene B through an unobserved pathway represented by the ‘Factors’ node. While these factors are unobserved we can still infer that there is a regulatory effect of Gene A on the downstream Gene B, which is represented in our network model by the parameter 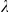.

Biologically, our goal is to identify relationships between the expression of multiple genes, such as the case depicted in Figure 1. In this figure we see that the expression level of Gene A has an effect on the expression level of Gene B, mediated through some biological process (i.e. unobserved factors). Even though we do not directly observe all the factors involved in the regulatory interaction, we still want to be able to detect that there is a regulatory effect, including the relative magnitude, the presence, and direction of the effect. To resolve these relationships uniquely, we need perturbations of expression, which in this case arise from genetic polymorphisms affecting expression. Therefore, both gene expression and genotype data needs to be collected on the same set of individuals, for all genes of interest, as well as all genotypes that will possibly act as perturbations of expression. Overall, one can consider our model selection process as acting on the joint covariance between and within the gene expression products and genotypes identified as being strong QTL. In our algorithm we further focus on *cis*-eQTL, because of recent studies indicating that there are widespread genetic polymorphisms local (i.e. *cis*) to genes that cause significant changes in expression [26]–[28].

We want to identify the genes with strong *cis*-eQTL (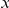) with linear effects on gene expression (
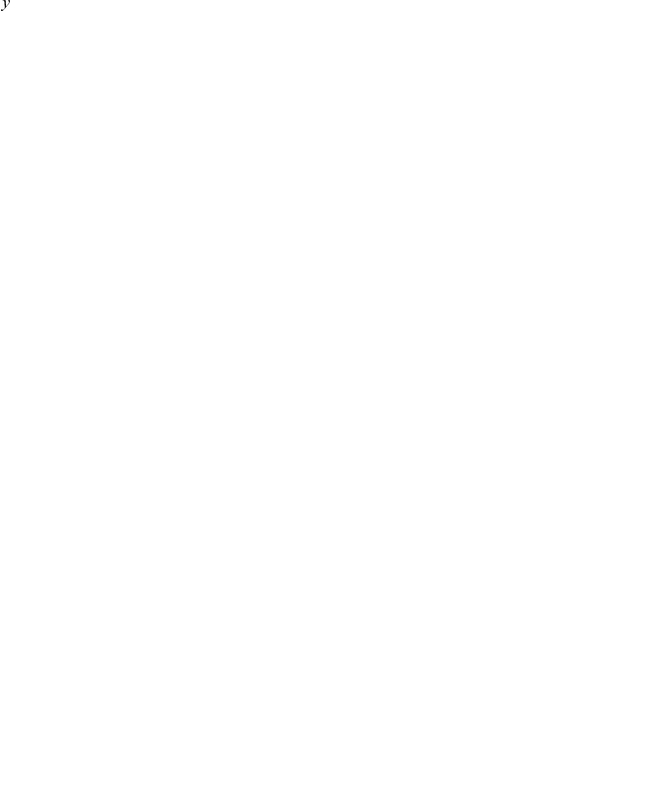
) parametrized by genetic effect parameters (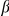), and then identify unique regulatory relationships among gene expression products parametrized by 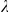. For 
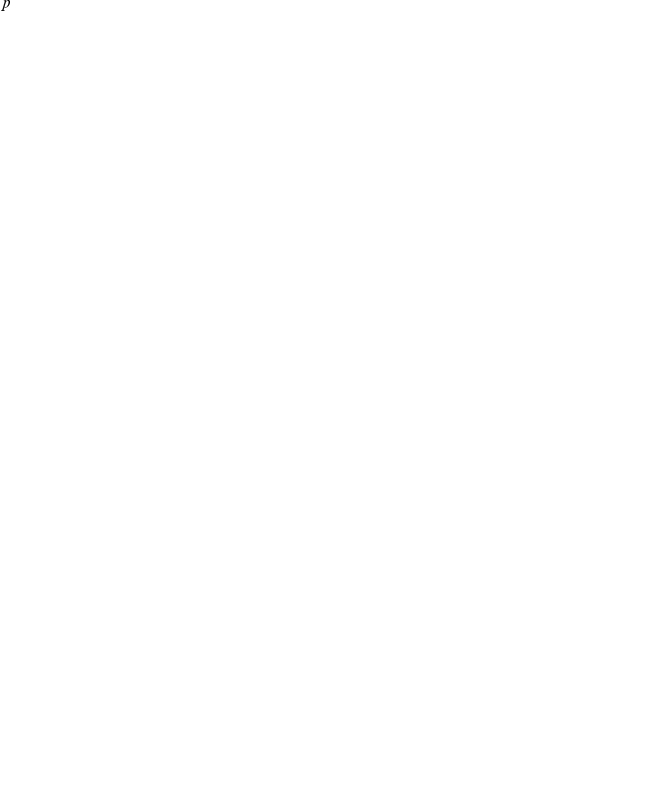
 measured gene expression phenotypes and 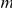 loci for which we have genotypes, the directed graphical model of the network has 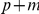 nodes and 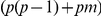 possible edges, representing 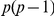 possible regulatory relationships among the genes, and 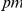 possible perturbation effects of loci (eQTL) on each of the expression phenotypes. Written in matrix notation, the network model for a sample of 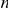 individuals can be represented as:
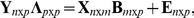
(1)where 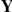 is a matrix of gene expression measurements, 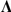 is a matrix of regulatory effects, 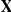 is a matrix of observed perturbations, 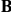 is a matrix of genetic effect parameters, and 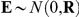, where 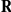 is a diagonal matrix. Non-zero elements of 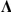 and 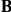 are edges representing regulatory relationships and eQTL effects, respectively, where the size of the parameter indicates the strength of the resulting relationship, as shown in Figure 1. Versions of this model are used regularly in analysis of networks [3], [8], [10] when assuming that gene expression measurements are taken from independent and identically distributed (*iid*) samples, where the regulatory relationships can be approximated by a system of linear equations, and the distribution of expression traits across samples is well modeled with a multivariate normal distribution. Another common assumption we make use of in our algorithm is that most detectable eQTL effects will have a significant linear component, especially for *cis*-eQTL [27], [28], where the polymorphism has simple switch-like behavior, such as determining whether transcription of the gene is up or down regulated.

**Figure 2:**
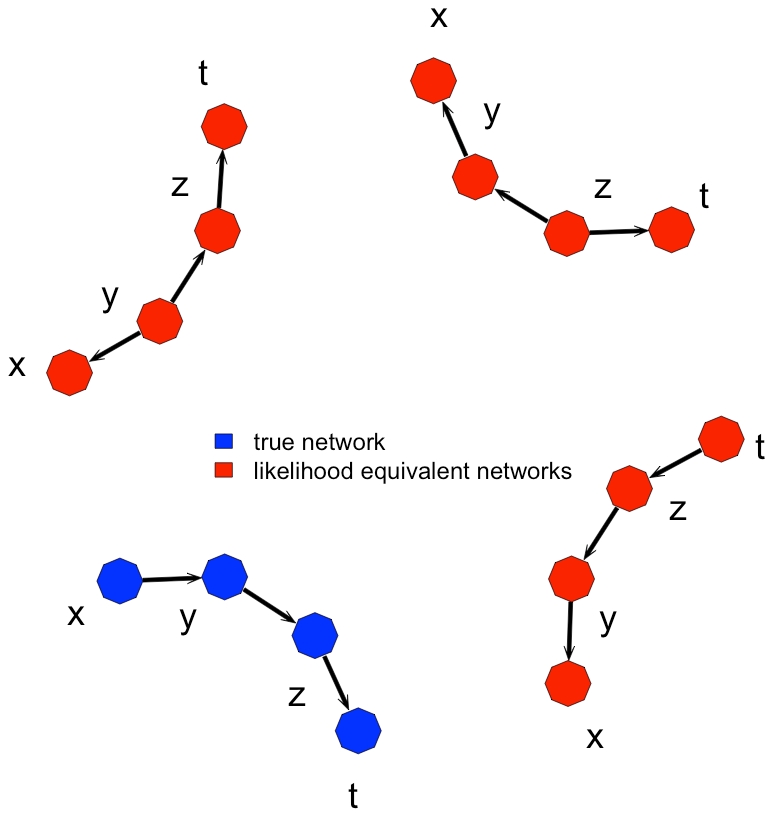
Example of a graphical model equivalence class when determining regulatory relationships among four genes (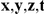). Edges represent the direction of regulation. In this case, the true regulatory network connecting the four genes (blue) has the same sampling distribution as the other three incorrect models (red). Without perturbations (i.e. eQTL), each of these models will equivalently describe the pattern of expression observed among these genes for any data-set.

A potential pitfall of modeling expression traits using directed networks of the type in Equation (1) is the problem of likelihood equivalence between models. Figure 2 presents a simple example that illustrates the problems raised by equivalence for network inference. In this example, the true model, which is a linear pathway between four genes 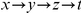, is indistinguishable from three other equivalent models. Each of these equivalent models has a very distinct implication for regulatory relationships among these genes but they are indistinguishable, regardless of the sample size. To be able to distinguish between these models, one needs to either collect time-course data to determine the temporal sequence in which regulation occurs [32], or alternatively, perturb the expression level of these genes in some fashion.

### The algorithm

**Figure 3:**
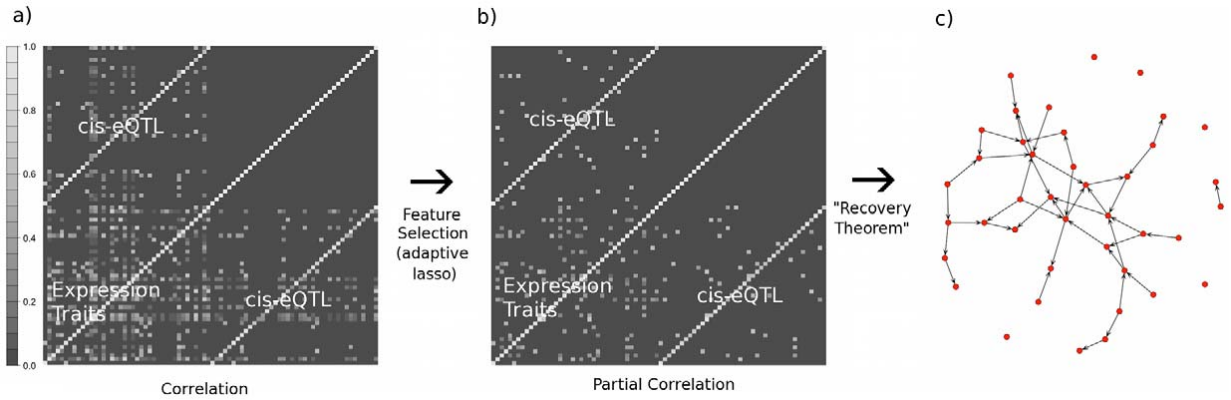
Outline of the structure of Step 2 of the algorithm. (a) After selection of phenotypes in Step 1, we produce a covariance matrix between observed gene expression products, and their associated unique *cis*-eQTL. (b) A convex feature selection method (the adaptive lasso) is used to learn the structure of the inverse covariance matrix, which is also the conditional independence or interaction network among gene expression products and *cis*-eQTL genotypes. (c) The directed cyclic network among expression products can then be recovered directly from the conditional independence network, using the “Recovery” Theorem. For Step 3, each of the induced edges between expression phenotypes and *cis*-eQTL, shown in (b), are tested to ensure marginal independence using a permutation test.

Our goal is to identify a unique network underlying the observed expression and genotype data, especially when the sample size is at most 1,000 (a large, biologically realistic sample size). To accomplish this, in the **Methods** we prove a set of theorems to show that if each gene being considered has its own, unique eQTL, then one can go from the sample covariance among gene expression phenotypes and genotypes (defined as **S** in the **Methods**, see Figure 3a), to the inverse covariance (i.e. precision matrix or undirected network defined as 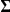 in the **Methods**, see Figure 3b), then subsequently to a directed cyclic network underlying the expression data (defined as 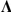, see Figure 3c), where the last step makes use of our “Recovery” Theorem. In the algorithm, we begin with a screening process to identify a set of expression traits with putative strong *cis*-eQTL (Step 1). We then make use of the adaptive lasso function for reconstruction of conditional independence networks (i.e. the structure of the inverse covariance matrix, Figure 3b) (Step 2) to identify genes with strong induced dependencies among *cis*-eQTL genotypes and gene expression phenotypes and reconstruct the unique directed acyclic or cyclic network that is a result of these induced edges. Finally, for each putative strong induced dependency, we further filter the induced edges based on a permutation test (Step 3), to ensure marginal independence between the upstream gene and the downstream *cis*-eQTL:

#### Step 1: Selection of expression phenotypes

A standard genome-wide association analysis is performed on each expression trait, focusing on genetic polymorphisms in a *cis*-window around a gene (e.g. a 1Mb window) [28]. Each marker is tested individually using either a linear statistical model or non-parametric test statistic (e.g. Spearman rank-correlation), with a correction for multiple tests using either a control of false discovery rate [33], a conservative Bonferroni correction (i.e. 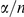, where 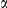 is the significance level and 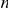 is the number of tests), or through a permutation approach to compute significance based on the empirical distribution of test statistics after shuffling the data, as in Stranger et al. [28]. After this initial association analysis is performed, the remaining *cis*-eQTL and their associated genes are further filtered such that the *cis*-eQTL genotypes are strongly independent of one another. In our analyses we use the very conservative cutoff 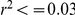 between any pair. This ensures that each *cis*-eQTL represents a unique perturbation, which is especially important for small sample sizes, when the sampling variability of the entire data-set is high.

#### Step 2: Regulatory network reconstruction

Once the set of expression phenotypes are identified, we combine the genotype and gene expression data, so as to infer a joint gene expression, *cis*-eQTL interaction network, (i.e. identifying which elements of the matrix 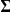 are non-zero). This model selection method is similar to the network recovery method proposed by [22], except using the adaptive lasso instead of the regular lasso [8]. The adaptive lasso procedure is performed by first solving the lasso problem:
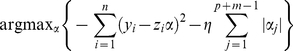
(2)then using the coefficients from this problem to solve the following adaptive lasso problem [25]:
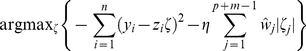
(3)for every phenotype, 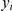 in the reduced data-set, where 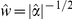, 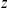 is the combined gene expression products and associated *cis*-eQTL genotypes, and 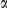 and 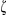 are the corresponding regression coefficients, whose non-zero structure should asymptotically be the same as 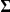, given an appropriate choice of the penalty parameter 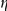. The penalty parameter 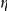 is chosen by five fold cross validation based on the mean-squared prediction error across both steps of the procedure. In addition, all variables are centered to have mean zero and rescaled to have variance one, so that the gene expression products and genotypes with small or large variances will not be penalized differently. After the interaction network is determined, we infer the directed regulatory network immediately from the interaction network structure, based on the results shown in the “Recovery” Theorem.

While we could make use of any undirected inference approach that infers the conditional independence network [11], [20], [29], [30] for Step 2, we use the adaptive lasso because of its theoretical advantages [25] and empirical performance, as far as finding sparse solutions with the lowest mean-squared error (by cross-validation) [8]. A lasso type procedure can be used for model selection [22] by shrinking parameters to exactly zero and is convex [34], providing computationally efficiency. However, there has been theoretical work showing that since the lasso shrinks non-zero parameters too harshly, it will not always return the true model asymptotically (i.e. as sample size goes to infinity). In fact the conditions under which it will return the correct model may be very unlikely for high dimensional problems [35]. The adaptive lasso was proposed to remedy this problem, and in general appears to have better properties as far as model selection both theoretically and in practice, without sacrificing the convexity of the lasso [8], [25].

#### Step 3: Edge interpretation and filtering

The primary goal of the “Recovery” Theorem is to map the problem of learning a directed cyclic graph among a set of phenotypes onto the problem of learning an undirected graph among a set of phenotypes and appropriately selected genotypes (i.e. unique *cis*-eQTL), then determining the corresponding directed cyclic graphs from the original problem. Each edge in this idealized larger undirected graph between the genotypes and the phenotypes represents an induced dependency between a given *cis*-eQTL and the immediate upstream phenotype of that *cis*-eQTL's *cis*-gene. Yet in practice, some of these edges identified in the undirected graph may arise from *trans*-effects, i.e. a given *cis*-eQTL may also have a large marginal correlation with another gene expression product in the data-set, that is not explained away entirely by the relationships inferred among phenotypes. In this case a further test can be performed, to ensure that for any putative induced dependencies identified from the undirected graph, the *cis*-eQTL and upstream gene are marginally uncorrelated. For this we perform a resampling method of the marginal correlation between *cis*-eQTL and upstream phenotype, and only use the edges which are very likely induced dependencies, in this case where the probability of observing a larger marginal correlation, given that they are uncorrelated, is 0.90. This threshold of 0.90 was used as a highly conservative threshold for marginal independence.

### Simulation analyses and comparison to other network recovery algorithms

To benchmark the performance of our algorithm, we compared it to the PC-algorithm [29], [31], the QDG algorithm [11], the QTLnet algorithm [16], and the NEO algorithm [18]. The other previously proposed cyclic algorithms either do not scale well (e.g. the approach of Li et al. [9]) or have prohibitively complex implementations (Richardson's cyclic recovery algorithm [20] or the algorithm of Liu et al. [10]). The PC-algorithm is designed to recover directed acyclic graphs using iterative tests of conditional dependence and independence, is a computationally efficient algorithm (scales to thousands of genes for sparse networks), and has competitive performance with other directed acyclic graph reconstruction algorithms [29], [36]. Additionally, the PC-algorithm forms the backbone of the QDG algorithm where it is used to construct an undirected graph (the skeleton of the directed acyclic graph) among expression phenotypes before orienting these edges using known QTL [11]. The QTLnet algorithm proposes a full Markov chain Monte Carlo approach to network inference, but does not scale above twenty phenotypes because of convergence rates of the Markov chain, and does not explicitly model directed cyclic graphs [16]. We also compared our algorithm to the NEO algorithm [18], and found that our approach controlled the false-discovery rate much better and had higher power for small networks (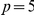, results not shown), but the implementation of the NEO algorithm available from the author was not stable for our simulations of larger networks (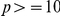), and so we did not include it in a larger comparison.

**Figure 4:**
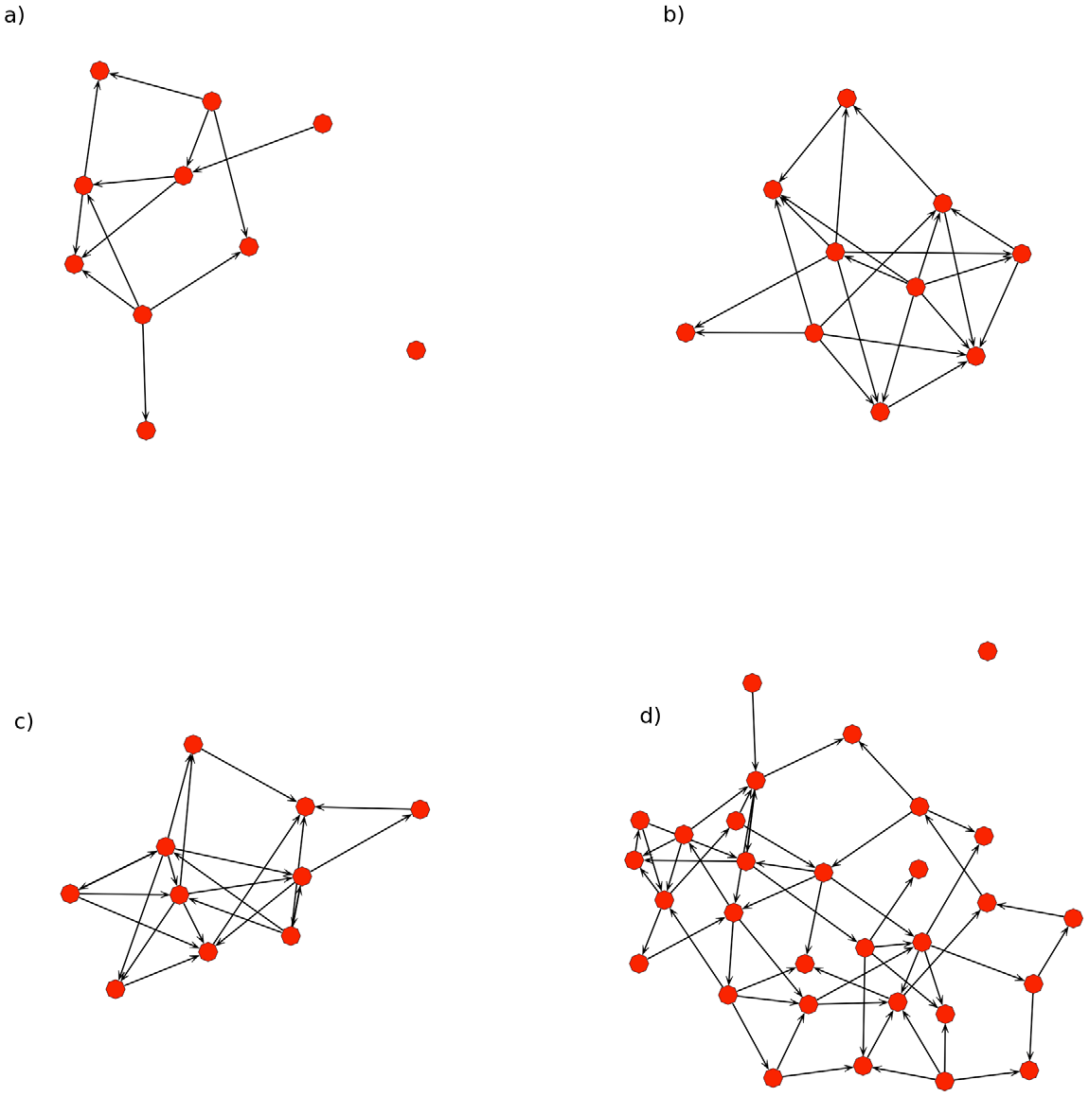
Examples of four network topologies used to simulate gene expression data from 160 total topologies. Sparse acyclic (a), dense acyclic (b), and dense cyclic (c) graphs were simulated for networks with 10 genes. Intermediately dense cyclic networks were simulated networks with 30 genes (d). Nodes represent expression levels of genes and the directed edges represent regulatory (conditional) relationships among genes, where the strength of the relationships were determined by sampling from a uniform distribution. Each phenotype (node) has a unique, independent cis-eQTL feeding into into it (not shown), with constant effect.

To compare the performance we simulated data from the model presented in Equation (1) with strong *cis*-eQTL, low sample variances, and different topologies, representing a scenario where there are strong eQTL, and few direct interactions between genes, with sample networks illustrated in Figure 4. The four different classes of simulations included directed acyclic graphs for 10 phenotypes, with sparse and dense topologies (Figure 4a, 4b), and directed cyclic graphs for dense (Figure 4c) and intermediate topologies (Figure 4d), with 10 and 30 phenotypes respectively, for a total of 160 distinct network topologies generated across all the simulations. This simulation is biologically motivated by the need for strong, statistically independent *cis*-eQTL and interactions among genes, as observed in previous studies [26]–[28].

We simulated a set of either 10 or 30 expression phenotypes and genotypes for sample sizes of 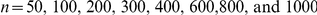 for both directed acyclic graphs and directed cyclic graphs. We simulated an F2 cross with the R package QTL [37], with either 10 or 30 independent known unique *cis*-eQTL of constant effect (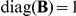), and error variances of 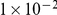. The regulatory effects (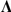) were sampled from a uniform distribution with parameters 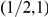 or 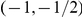 with equal probability. The network topologies were generated by randomly connected variables with equal probability, where the expected number of edges for each variable was either one, two, or three.

**Figure 5:**
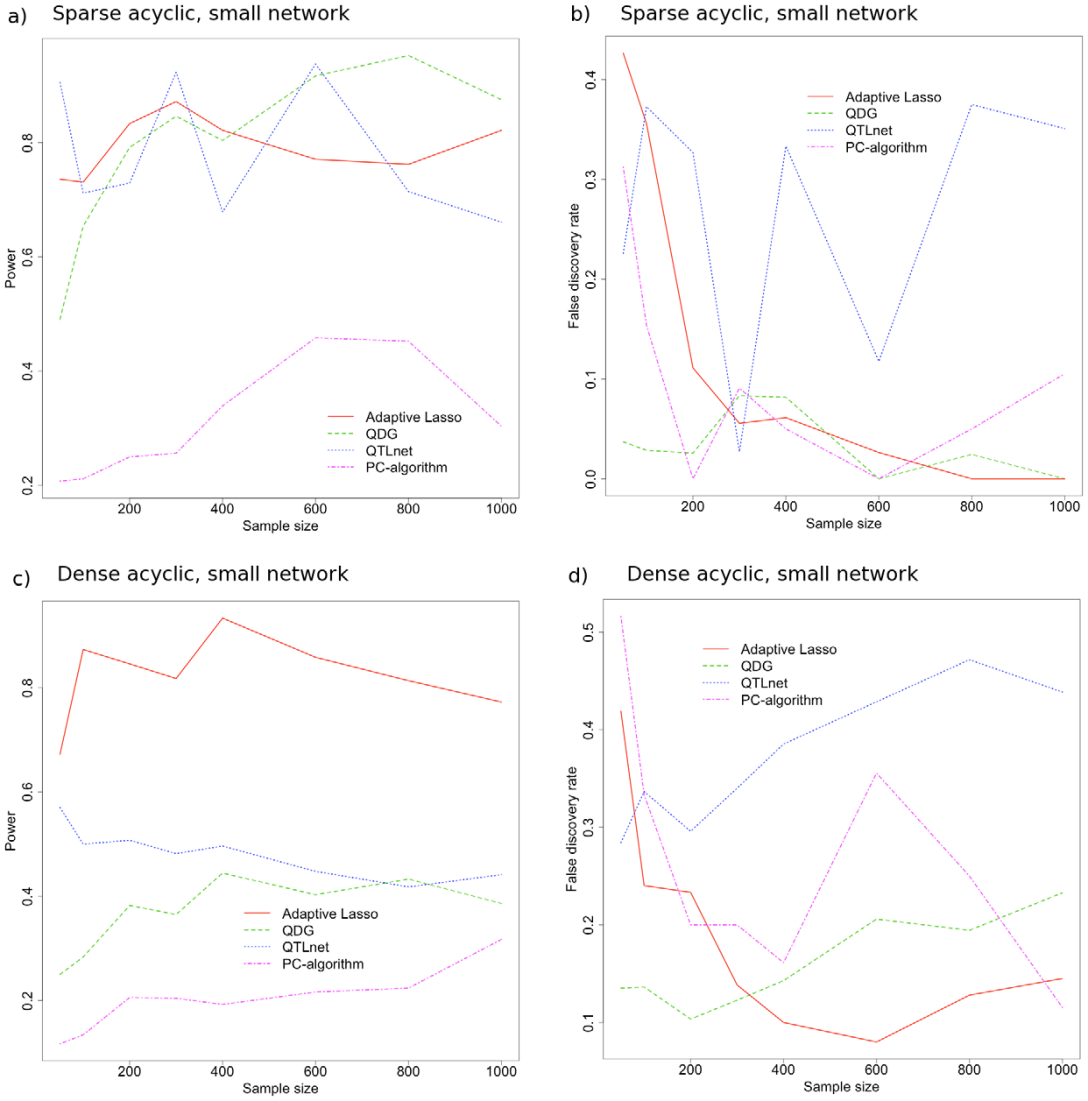
Performance of our algorithm using the adaptive lasso for directed acyclic graphs compared to other algorithms. These other algorithms include the PC-algorithm, the QDG algorithm, and the QTLnet algorithm for reconstructing different acyclic topologies of 10 genes. For a sparse directed acyclic topology (as in Figure 4a), the power (a) and false discovery rate (b) are plotted as a function of the sample size for five replicate simulations. Similarly, for a dense directed acyclic topology (as in Figure 4b), the power (c) and false discovery rate (d) are plotted.

**Figure 6:**
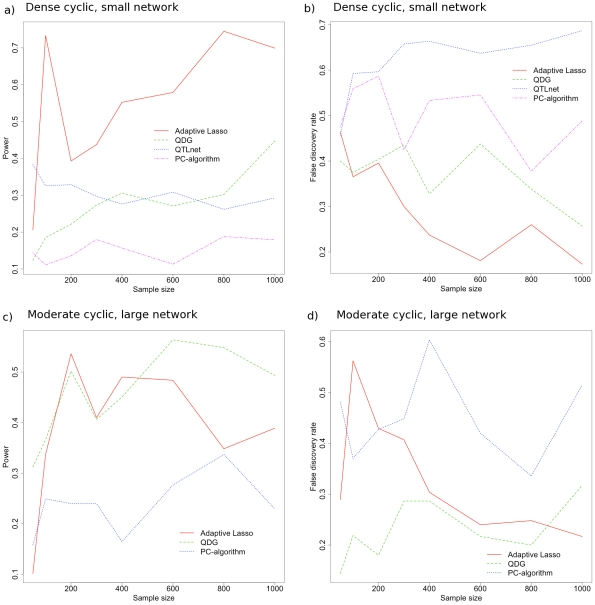
Performance of our algorithm using the adaptive lasso for directed cyclic graphs compared to other algorithms. These other algorithms include the PC-algorithm, the QDG algorithm, and the QTLnet algorithm for reconstructing different cyclic topologies of 10 genes (a) and (b) or 30 genes (c) and (d). For a dense directed cyclic topology (as in Figure 4c), the power (a) and false discovery rate (b) are plotted as a function of the sample size for five replicate simulations. Similarly, for an intermediately dense directed cyclic topology of 30 genes (as in Figure 4d), the power (c) and false discovery rate (d) are plotted.

Five replicate simulations were performed, sampling a new network topology and parameterization each time, and the power and false-discovery rate were computed for the adaptive lasso, PC-algorithm, QDG algorithm, and QTLnet algorithm for 10 expression traits, and all except QTLnet for 30 expression traits (because of the scaling of QTLnet). In addition, because we simulate the QTL independently, with no *trans* effects, we do not perform the third step of our adaptive lasso algorithm. We compared the performance for both directed acyclic graphs as well as directed cyclic graphs. In Figure 5 and Figure 6 we show the power and false discovery rate for recovering the correct set of directed edges using these methods. While some of the power and false-discovery rate curves show large fluctuations with increasing sample size in Figure 5 and Figure 6, this is due to elevated sampling variability due to each replicate simulation having a unique topology and parameterization.

For two of these scenarios, we show that our algorithm using the adaptive lasso can outperform the PC-algorithm, the QDG algorithm, and QTLnet in terms of statistical performance (see Figure 5c, 5d and Figure 6a, 6b) with similar computational scaling. In general, only the QDG algorithm has competitive performance with the adaptive lasso (see Figure 6c, 6d). This indicates that the necessary sample size to have a significant performance gain over the QDG algorithm may be much larger than is biologically realistic for larger more complex networks. These are significant results in two ways, the first being that we show that a feature selection method using linear regression can 1) identify directed regulatory architecture (given sufficient perturbations) and 2) it can also outperform state of the art network reconstruction algorithms, given a sufficient samples size and appropriate model dimension.

The adaptive lasso approach appears to work the best for smaller problems (i.e. 10 phenotypes) with denser topologies (i.e. Figure 4b, 4c) and performs better than other approaches in such cases (see Figure 5c, 5d and Figure 6a, 6b). This may be because smaller dimensional problems behave asymptotically at a faster rate. Unfortunately, this suggests that for larger problems (e.g. hundreds to thousands of phenotypes), unless the true topology is relatively sparse, the adaptive lasso, and perhaps all of these approaches will have poor performance without unrealistically large sample sizes (e.g. thousands) for both directed acyclic and cyclic graphs. We also performed a simulation for a small network (e.g. 10 phenotypes and 10 *cis*-eQTL), with dense directed acyclic topology and 200 or 1000 individuals with random variances and eQTL effects simulated from a 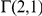 distribution. We found a uniform reduction in power (10–20%) across all methods, as well as a modest increase in false discovery rate (5–10%). Increased sample size appeared to correct for this additional randomness in the parameterization (results not shown).

### Yeast network analysis

We used our algorithm to reconstruct network structure for genome-wide gene expression data and genetic markers assayed in 112 segregants of a cross between two strains of *Saccharomyces cerevisiae*, reported by Brem and Kruglyak [27]. This cross was between a lab strain (BY4716) and a wild strain (RM11-1a), with 2,957 genetic markers genotyped and expression levels for 5,727 genes measured. While the sample size is relatively small, the study was well powered, with many strong *cis*-eQTL and interactions among genes [27]. An individual marker analysis was run around the *cis* region of each gene (25 kb around the start site of the gene) to identify a set of gene expression products with strong *cis*-eQTL (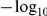
(p-value)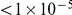), which identified 262 genes. We further filtered this set to remove *cis*-eQTL genotypes with high linkage, by filtering for a set with pairwise 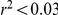 between any two *cis*-eQTL genotypes. Additionally, we tested the robustness of the inferred edges by randomly sampling the flanking genetic markers 20 times for all *cis*-eQTL and refitting the model. The percentage recovery for the top six recovered directed edges for the 20 resamplings are shown in **Table 1**. All missing data for a given genotype or phenotype was set to the sample mean of the respective variable.

**Table 1:** Directed regulatory edges identified by the adaptive lasso for *S. cerevisiae* cross.

Regulator gene	Target gene	Scaled effect	% Recovery from adjacent marker resamplings
TYR1	RCY1	0.035	0.05
TYR1	JLP1	0.123	0.35
TYR1	BUB2	−0.0056	0.55
TYR1	PRM7	0.0576	0.55
SEN1	MST27	−0.135	0.85
PRM7	POC4	0.154	0.15

**Figure 7:**
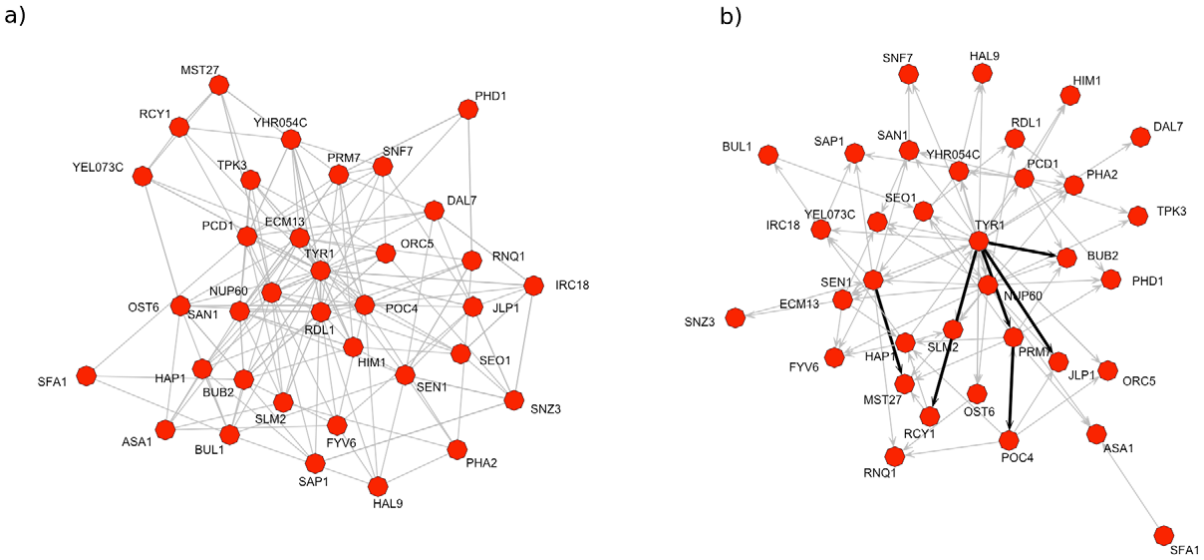
Sparse network reconstruction among 35 gene expression products. These genes were filtered for having strong, independent *cis*-eQTL (pairwise 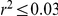) using the adaptive lasso algorithm for a *Saccharomyces cerevisiae* cross between a wild strain and lab strain [27], with 112 segregants (see text for details). (a) Recovered undirected network among these 35 gene expression products and (b) putative directed network reconstructed for the same genes, based on the edges between *cis*-eQTL (not shown) and the 35 genes. Bold edges represent directed edges with strong confidence based on a resampling procedure (see text for details).

After the additional filtering described above, we were left with 35 genes with unique, independent *cis*-eQTL, with an undirected network shown in Figure 7a, and possibly directed network shown in Figure 7b. Performing the adaptive lasso procedure on these 35 gene expression phenotypes and 35 genotypes identified 91 possibly directed edges among these genes, and 145 undirected edges among the genes. These hits were further filtered to ensure they represented induced dependencies, leaving six edges with relatively strong evidence of directionality (see **Table 1** and Figure 7b). These include four edges feeding out of the TYR1 gene, a gene involved in tyrosine biosynthesis [38]. Since TYR1 is also a hub in the undirected network (see Figure 7a), this suggests that amino acid biosynthesis, and perhaps anabolism in general is driving the expression of many of this particular subset of genes. The genes in which TYR1 appears to have direct effects on have diverse molecular and biological functions including endocytosis (RCY1), sulfonate catabolism (JLP1), cell-cycle checkpoint (BUB2), and cell-cell communication (PRM7) [39]–[42].

Additionally PRM7 feeds into POC4, a proteasome chaperone protein [43], representing possible cross-talk between cell-cell communication response and protein processing. Finally, SEN1, a helicase indicated in RNA polymerase 2 termination [44], appears to robustly directly affect MST27 an integral membrane protein implicated in vesicle formation [45]. In the implied undirected graph, there were striking topological features, including an average degree of 8.28 (relatively dense), and four genes appeared to be major hubs of a sort, TYR1, NUP60, RDL1, and POC4. These hub genes may represent major axes of variation driving the expression of this subset of genes including processes such as amino acid biosynthesis, information transfer across the nuclear envelope [46], and protein degradation. While most of the edges in the network were not orientable, there still appeared to be many dependencies (even with a possibly high false-discovery rate), indicating a potentially complex set of regulatory interactions, projected on this subset of genes, driving variation in expression. Additionally, there were many edges from eQTL that would appear to be *trans* associations (i.e. with large marginal correlations), demonstrating that many of the pathways that mediate these *trans* genetic effects are not captured in the observed sets of genes. Based on the simulation study, and the complexity of the recovered network (which most likely indicates a high false discovery rate), a much higher sample size would need to be collected to definitively resolve this possible set of regulatory interactions, and have increased confidence in the directional interpretation of the induced edges.

## Discussion

Our algorithm represents a novel approach to directed network recovery by making use of a convex optimization approach for regulatory feature selection when analyzing gene expression products and *cis*-eQTL. This is the first algorithm that makes use of sufficient sets of *cis*-eQTL to infer unique directed cyclic networks from gene expression data with a feature selection methodology. Our use of the adaptive lasso procedure for feature selection has significant computational and theoretical advantages, since the underlying optimization program is convex (ensuring a computationally efficient, unique solution), is model selection consistent, and has the oracle property (asymptotically, the estimates of the non-zero regression coefficients behave as if the model was known *a priori*) [25]. There have not been many algorithms proposed for genome-wide cyclic regulatory network recovery, [9]–[11], [20] and they all have either computational or theoretical challenges associated with them, including heuristic searches through regulatory network space with no guarantee to reach networks with the strongest evidence given the data [10], [11], [18], or lack sufficient perturbations to allow unambiguous regulatory inference [9], [20]. With respect to directed acyclic network recovery, we see in the simulations that our feature selection approach with sufficient perturbations outperforms the PC-algorithm, the QDG algorithm, and the QTLnet algorithm for dense, small scale problems as shown in Figure 5c, 5d and Figure 6a, 6b. This increase in performance is a direct function of the adaptive lasso procedure correctly identifying the children of a given node, which will then force an edge to appear between the additional co-parents of that node, and its unique cis-eQTL. Once all these induced edges are identified, the structure of the directed network can be elucidated, since all the expression parents of each gene will be known. Our algorithm also does this all in a single optimization procedure, avoiding sets of iterative tests, where type-I and type-II errors can build up at each stage, such as in the PC-algorithm. Alternatively for larger more complex graphs the performance appears to be similar to that of the QDG algorithm Figure 6c, 6d, perhaps because the asymptotic properties take much larger sample sizes to be practically realized.

For the analysis of the yeast data the topology of the identified network included many undirected cycles, with the few orientable edges being acyclic, as shown in Figure 7. In addition there were a set of genes which appeared to be hubs (the most connected being TYR1, NUP60, RDL1, POC4, and SEN1, PCD1, and SAN1 to a lesser extent). This phenomena is probably in part due to an inflation in false-positives because of the small sample size, and a complex underlying model with many unobserved variables. Yet a subset of these edges may represent hub genes capturing different broad patterns of variation across this entire sub-network. Even though most of the edges in this network are not orientable, an experiment could be devised where each of these hubs was perturbed, and given the topology it would produce a prediction about how a relatively large set of other genes in the hub's neighborhood would behave. More strongly, in the case of the TYR1 gene which had the most orientable edges, it suggests that if the process driving that gene's expression was stopped, many other genes would also be affected, but not vice-versa.

A number of assumptions concerning biological networks are implicit to our algorithm. These include assumptions that are common to most graphical modeling techniques, such as sparsity, faithfulness, linearity of regulatory relationships, and normally distributed error, as well as an assumption that is specific to our algorithm: the presence of known, independent perturbations from *cis*-eQTL. The common assumptions are reasonable when constructing a first approximation to regulatory network structure. Sparsity and faithfulness (i.e. the true network does not contain pathological parametrizations where there is parameter cancellation) are essential assumptions that are implicit in algorithms for both directed and undirected network inference algorithms [5], [6], [11], [16], [20], [29], [30]. Regulatory relationships are not linear, but linearity is the simplest approximation that provides biologically relevant information, i.e. there is a detectable relationship between two genes, or no relationship. An assumption of normality is conservative in terms of being the most ‘random’ distribution that could have generated the data, since given an observed covariance structure, normal distributions have maximum entropy [47]. Given the absence of knowledge about the specific biological process generating the distribution of expression measurement error, and barring any clear evidence of non-normality in data, such a conservative approximation is appropriate.

The assumption of independent, detectable *cis*-eQTL effects is the most restrictive assumption. Other methods have proposed to use *trans*-eQTL directly to increase the power to detect causal relationships and reduce the space of equivalent models [5], [9]–[11], [16], [18], [19]. We require the assumption of only *cis*-eQTL, because without it, there is no longer the exact isomorphism between the undirected graph among genotypes and phenotypes and the directed cyclic graph among phenotypes. This occurs because in the case of directed cyclic graphs, it is statistically impossible to know which phenotype in a network a *trans*-eQTL directly feeds into, unless their is prior knowledge about the true causal structure of the system, as with the assumption we make about *cis*-eQTL. This statistical degeneracy arises as a result of the “Recovery” Theorem, where when there is a set of equivalent models with independent, unique perturbations, that contains reversals of cycles, each equivalent directed cyclic graph will have an alternative perturbation topology (i.e. the mapping between unique eQTL and gene expression phenotypes, determining which eQTL causally affects which gene expression product).

Alternatively, as we show in real data, even if there do appear to be many *trans*-eQTL we can still detect a subset of edges from the *cis*-eQTL that behave how we would like (by using Step 3 of the algorithm). While this may reduce our power to detect directed cycles in practice, it ensures that for real data-analysis we are more confident in the edges we reconstruct. Another possible solution to the incorporation of *trans*-eQTL would be to use the adaptive lasso to generate the initial undirected graph among genotypes and phenotypes, then to orient the edges in the graph using an iterated testing approach, as in the NEO algorithm [18], the algorithm of Millstein et al. [19], or the QDG algorithm [11]. We do not expect the requirement of unique *cis*-eQTLs to be a good approximation for all regulatory modeling situations. However, this assumption also seems reasonable, given recent biological observations of strong local polymorphism associations with gene expression (eQTL) which are often not in linkage disequilibrium [[26]-, [28], [48], [49]]. What is more, due to the structure of linkage disequilibrium in outbred populations (the correlation structure among genotypes) it is often possible to identify a large set of *cis*-eQTL that are uncorrelated and each have unique expression phenotypes, e.g. a set of eQTL that are present on different chromosomes or are far away from one another in terms of genetic map distance [28].

As a final comment, the theory of sufficient perturbations that maximize regulatory resolution, which is used as the foundation of our algorithm, is quite general, and could be used to integrate multiple data types to make predictions about putative causal regulators underlying complex phenotypes, such as disease [1], [2]. The “Recovery” Theorem defines a class of perturbation architectures where there is a direct isomorphism between two very different types of networks: the inverse covariance structure (an undirected network) with perturbations and a directed cyclic graph representing a regulatory network. The theory does not require perturbations to be *cis*, just that there be an appropriate set of perturbations that provide resolution. More complex perturbation sets, which include sufficient perturbations as a subset, can also provide maximum resolution. One could therefore construct algorithms similar to the algorithm presented in this paper, without the local *cis* perturbation restriction. Moreover, the specific topology of eQTL effects need not be known, if one is willing to accept the cost of larger network equivalence classes and therefore less total regulatory resolution. With this restriction lifted, it would be possible to jointly infer the genetic perturbation architecture simultaneously with regulatory architecture, although such a joint reconstruction would require much larger sample sizes.

## Methods

### The network model

The network model is presented in equation (1). For this model, we make the assumption that in the true network model, 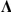 is sparse. In addition, we assume that 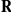, the error covariance matrix of expression products, is diagonal, and 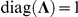, where the constraint on the diagonal of 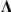 ensures model identifiability. This constraint corresponds to a lack of self-loops, since the parameters representing self-loops are confounded with the error variance parameters specified by 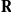. These latter assumptions on 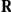 and 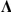 (i.e. no error covariance or self-loops) are standard, and used by all popular graphical network inference algorithms, directed and undirected, proposed to date [3], [6], [9]–[11], [14], [20], [29], [31]. The model depicted by Equation (1) is a completely observed structural equation model (SEM) [50].

### Likelihood and equivalence

The conditional log-likelihood of the model defined by Equation (1) can be written as:
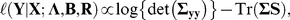
(4)where the full precision matrix 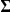 and empirical covariance matrix 
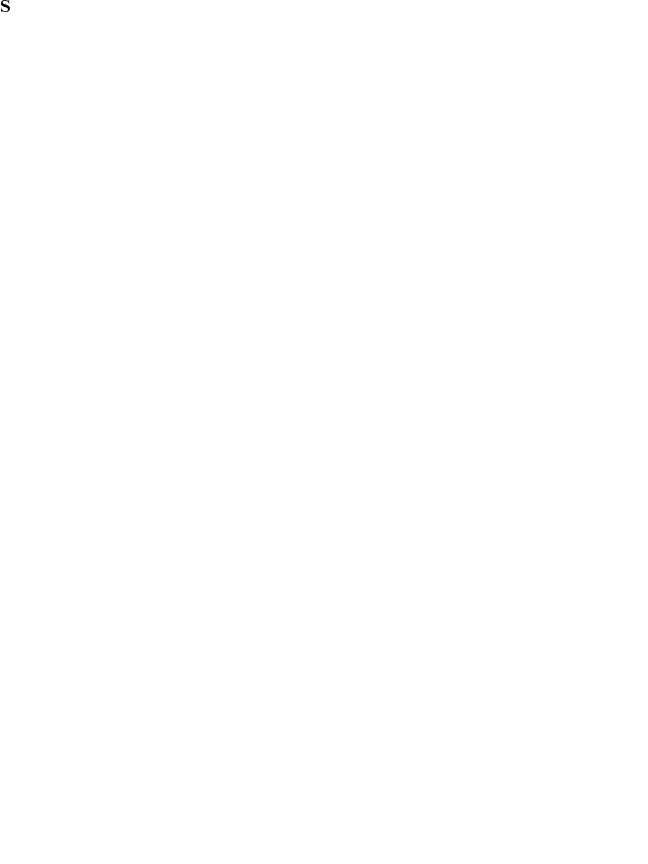
 are:
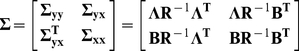
(5)

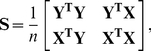
(6)with the data matrices 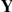 and 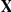 re-centered.

We can define a fully parametrized model matrix 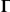:
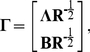
(7)since by definition 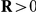, and 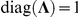, both 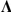 and 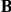 can be rescaled by the positive square root of the error precision matrix 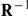.

From Equation (5), Equation (6), and Equation (7) the relationship between the fully parametrized model matrix 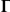, and the full precision matrix 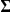 is
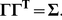
(8)This defines a system of homogeneous polynomials of degree two which exactly specifies the relationship between the directed graph 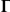, which may contain no cycles (a directed acyclic graph or DAG) or may contain cycles (a directed cyclic graph or DCG), and the moralized undirected graph 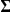.

#### Definition of equivalence [51]

Two sparse directed cyclic graphs specified by the model in Equation (1), with parametrization 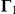 and 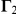, are equivalent in distribution iff for all parametrizations 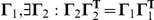 and for all parametrizations 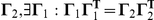.

Intuitively, the parametrization defined by 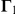 and 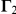 provide a unified representation of the directed cyclic graph among gene expression products along with the set of perturbations of expression (i.e. genotypes). This definition of equivalence allows us to characterize our theory of sufficient perturbations.

### “Recovery” theorems

Given the importance of having as small a set of equivalent models as possible for making meaningful inference, and the necessity of perturbations for minimizing equivalence classes, it is of interest to know what will constitute a sufficient set of perturbations, i.e. to shrink the size of arbitrary equivalence classes as much as possible. In the following section we provide proofs of three theorems that describe such a set. We note that it should also be possible to use the work of Richardson on cyclic causal discovery [20] to arrive at the same theoretical condition concerning a set of sufficient perturbations, though it is beyond the scope of this work to show this connection. Here, we use an independent and simpler proof based on normal theory and matrix algebra. Our theory also provides a generalization of the work of Chaibub Neto et al. [11], which shows that sets of unique (or “driving”) QTL for each phenotype can be used to uniquely orient edges in a directed cyclic network. Our approach allows us to represent the problem of directed network inference as a model selection problem within a regression equation for each phenotype. This allows us to avoid the reliance on computationally inefficient heuristics [3], [10], [11], which can generate many possibly poor-fitting networks depending on how the algorithm is run, when considering sample sizes that are typical of experiments collecting genome-wide gene expression data.

The “Recovery” Theorem demonstrates how the set of equivalent DCGs can be recovered from the precision matrix between expression phenotypes and loci (the matrix 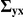). This last result is incorporated into our algorithm for inferring sparse network structure with a sufficient perturbation (eQTL) set. Note that while the algorithm depends on sparsity for efficient network recovery, the results of these theorems are general and do not require such a constraint. In addition, we note in a further **Lemma** that even in the case of directed cycles, if we know which phenotype a perturbation feeds into, we can further reduce the size of the equivalence class to a unique directed cyclic graph.

**Theorem 1:** Given two distribution equivalent directed cyclic graphs, with equivalent parametrizations 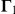 and 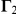, any matrix **A** which satisfies 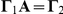, must be orthonormal (i.e. 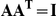).

**Proof of Theorem 1:** Since 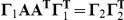, and from the definition of equivalence, if 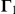 and 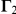 are equivalent, then 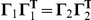. Therefore, 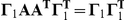. Left multiply by 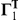 and right multiply by 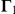, then 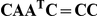, where 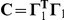 is a positive definite invertible matrix of rank 
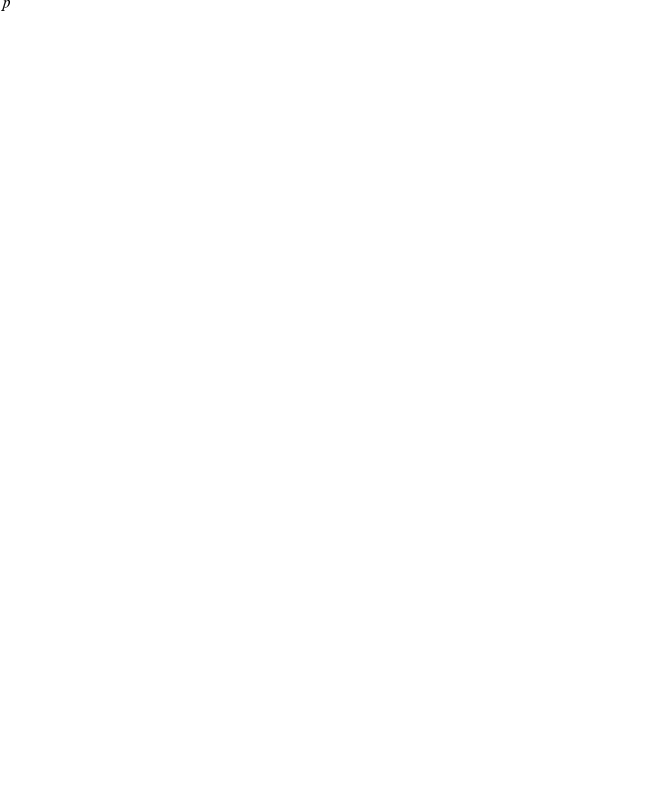
. Left and right multiply by 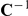, and 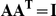.

The matrix 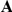 can be thought of as a linear operator that allows transformations between models which produce the same covariance (and inverse covariance) structure (even between models which are not faithful). We use this operator to prove the following theorem after rescaling the network and perturbation parameters as in Equation (7): 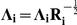, 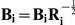:

**Theorem 2:** If there exists an ordered set 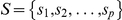 of rows of the perturbation graph parametrized by 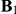 such that 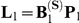, where 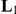 is a diagonal matrix of rank 
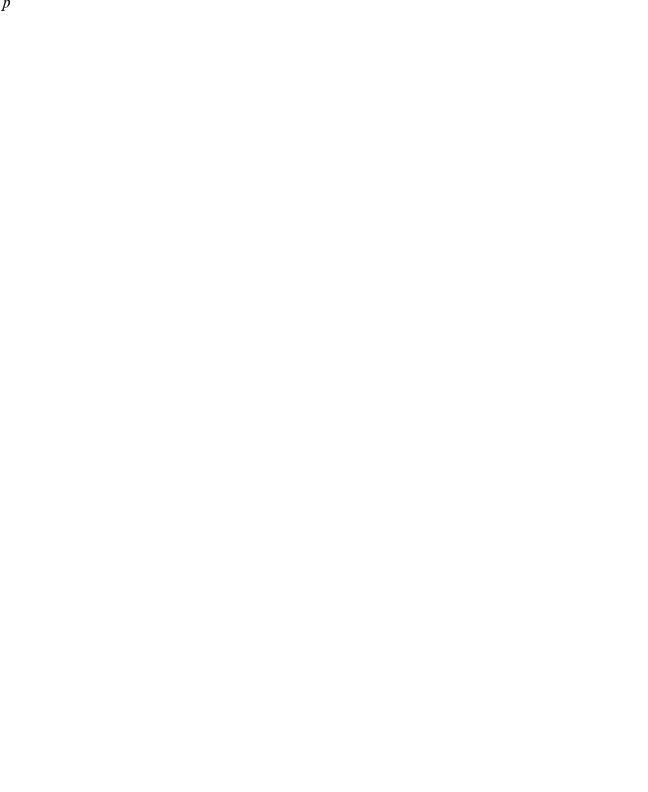
 and 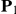 is a signed permutation matrix, then 1) if 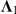 parametrizes a DAG, then for any parametrization 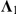 of any DAG, there does not exist an alternative equivalent DAG or DCG, and 2) if 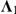 parametrizes a DCG, then for any parametrization of any DCG, there exists a finite set of equivalent DCGs, where each equivalent DCG contains a reversed directed cycle with reference to the original DCG.

**Proof of Theorem 2:** Given 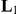 exists, assume there exists an alternative equivalent model parametrized by 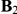 and 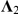. Then, by Theorem 1, there exists an orthonormal matrix 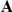 where 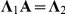, 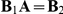, and 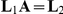. Because 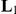 and 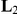 are invertible, we have: 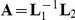. This implies that 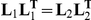. Since 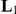 is diagonal for any parametrization 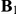, 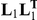 and 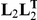 must also be diagonal for all equivalent parametrizations 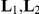. If there does not exist a signed permutation matrix 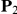 such that 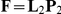, with 
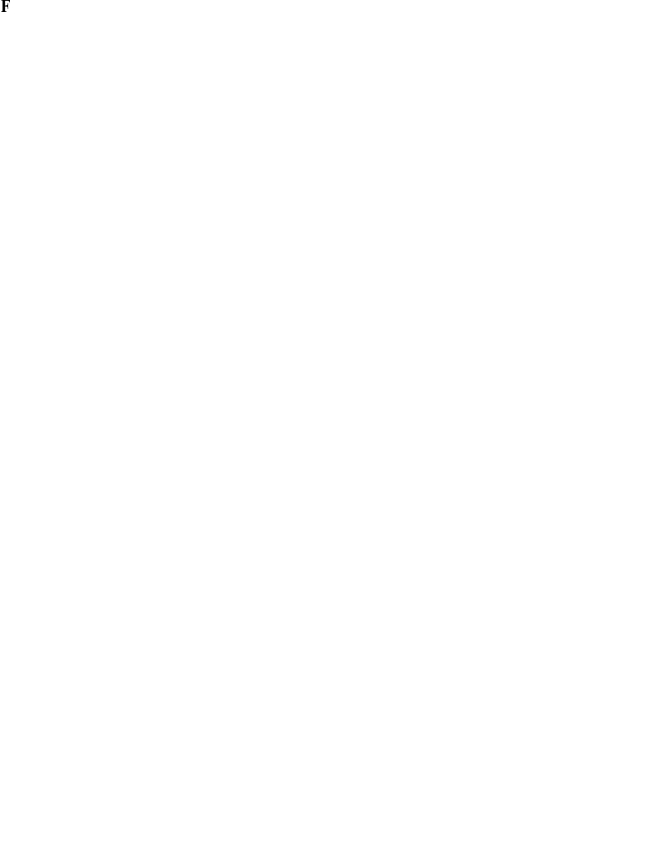
 diagonal, then there always exists a parametrization of 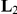 where 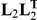 is not diagonal, and therefore not equivalent (since all non-zero elements of 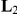 are free to vary). Therefore 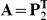 is either an identity matrix or a signed permutation matrix. Now consider 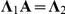. Because in this parametrization, 
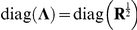
, the only allowable equivalent model transformations must have positive non-zero elements along the entire diagonal. Therefore, if 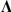 parametrizes a DAG, then 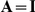, and if 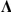 parametrizes a DCG, then 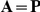 where 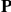 is any signed permutation matrix which ensures non-zero positive elements along the diagonal of 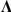. This corresponds directly to reversing the order of any set of directed cycles in the graph.

This theorem allows us to understand constraints on possible equivalent models in the specific case when each node has at least one unique perturbation. In the next theorem, we focus on the structure of the moralized graph (i.e. the precision matrix 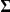) for these models, and see how it maps back to the set of possible unmoralized directed graphs that generated the moralized graph. We define the set of parents of a particular node, 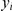, from the directed graph as 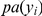, and the set of all nodes in an undirected graph 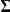 that have edges to node 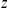 as 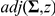.

**“Recovery” Theorem:** If in 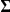 there exists an independent perturbation vertex set 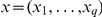 and a response vertex set 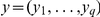 where 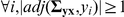 and 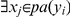, then the only equivalent directed cyclic graphs among 
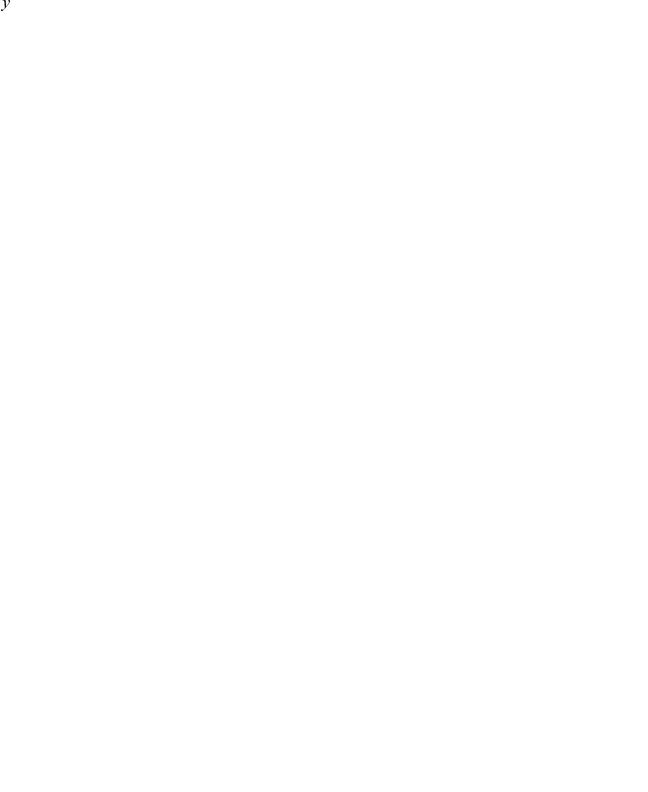
 that could have generated 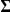 contain permutations of cycles, and can be recovered from 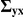.

**Proof of the “Recovery” Theorem:** The existence of an independent perturbation vertex set and response vertex set that satisfies these conditions corresponds directly to a perturbation topology and parametrization specified by 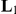 from Theorem 2. Given this observation, Theorem 2 ensures the constraint on possible equivalent models. Finally, the reason the structure can be recovered from 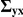 is apparent from Equation (5) and (7), where 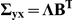, and therefore 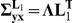 Since 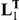 is diagonal it won't change which elements of 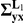 are zero or non-zero.

In the case of DAGs, a generalization of this theorem is trivial to prove for graphs defined over arbitrary probability measures, since the process of moralization of a graph connects all the parents of a given node. Since in this specific perturbation case, each node has at least one unique parent (from the perturbations), then a connection will be induced between the unique perturbation parent and each of its co-parents, indicating exactly what the unique set of parents are for that given node.

Alternatively, as we saw in Theorem 2, the assumptions of normality and linearity are key to showing that even for directed cyclic graphs that have unique perturbations, there still exists multiple equivalent models. In the “Recovery” Theorem we see that we can still determine these ‘minimal’ equivalence classes from the moralized graph. It is interesting to observe that the perturbation topology can completely change among equivalent directed cyclic graphs, whereas it cannot for directed acyclic graphs. If one knows which node each perturbation feeds into, then the following is true:

**Lemma:** If the underlying perturbation topology, 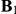, is known, then the cardinality of all directed cyclic equivalence classes is reduced to one.

This further reduction of the equivalence relationships is apparent when one considers that each equivalent perturbation topology specifies exactly one member of the equivalence class (from the “Recovery” Theorem). Therefore, if one knows the true perturbation topology, then one knows the true regulatory model. This allows us to infer a unique directed cyclic graph in the case where we know which phenotype each genetic perturbation feeds into. Hence, the reason behind making our major biological assumption: to only consider the genetic effects of *cis*-eQTL and assume that the *cis*-eQTL feeds directly and uniquely (i.e. non-pleiotropically) into the local gene. With *trans*-eQTL, unless there is prior knowledge about exactly which gene each *trans*-eQTL affects (i.e. about the pathways in question), there is no way to reduce this equivalence class to a unique directed cyclic graph.

### Algorithms

**Adaptive lasso**. For Step 1 of the algorithm, we perform an individual marker analysis of each genetic polymorphism in a window around the start site of the gene, and only include the markers that are significant given a Bonferroni correction for multiple testing. We then filter these sets of *cis*-eQTL such that they are effectively independent given the linkage disequilibrium structure of the data. For the analysis of the yeast data, we found that a maximum pairwise 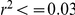 between *cis*-eQTL genotypes was a very conservative threshold given a resampling test of random markers across the genome (results not shown).

For Step 2 of the algorithm, the lasso problems from Equation (2) and (3) are solved using the cyclic coordinate descent method of Friedman et al. [52], as implemented in the ‘glmnet’ package, called by the ‘parcor’ package [8]. While this method is an approximation to solving the adaptive lasso for the log-likelihood defined in Equation (4), there are theoretical connections between an exact solution to the problem, and this approximate solution which suggest that in some cases the approximation will not perform much worse than the exact solution (i.e. highly penalized cases) [23].

For Step 3 of the algorithm, we performed a permutation test to very conservatively ensure that the induced edge found between an upstream gene, and the *cis*-eQTL, did not arise from a *trans*-effect of the *cis*-eQTL. To do this we randomly resampled the genotype data 10,000 times for each induced edge, and determined the proportion of the time the absolute value of the marginal correlation between upstream gene and *cis*-eQTL under the empirical null model was greater than the absolute value of the observed marginal correlation. We only treated induced edges as representing a directed relationship between a pair of phenotypes if the probability of observing a greater value under the empirical null model was greater than 0.90.

**PC-algorithm**. While this is only designed to reconstruct directed acyclic graphs, it has been used in a combined gene expression and genotype context to reconstruct directed cyclic graphs [11]. The PC-algorithm reconstructs the skeleton (i.e. set of edges regardless of edge orientation) of a partially directed acyclic graph (PDAG) by performing forward tests of conditional independence. It first starts by constructing a correlation graph (i.e. a conditional independence graph where one conditions on the empty set), then in a forward step-wise manner, removing edges in the neighborhood of each node by increasing the size of the conditioning set based on the neighborhood of each node. Once the cardinality of the conditioning set is equal to or larger than the neighborhood for all nodes, the algorithm terminates. While this is being done, all identified v-structures (co-parents of a common child) are being tabulated, so that afterwards these edges can be oriented. Then, there is a set of rules, based on the seed v-structures which orient a small initial set of edges, which orient many additional edges in the network, by propagating the implications of the few initial oriented edges, with respect to the d-separation criterion defined for directed acyclic graphs [29], [31].

We applied the PC-algorithm by giving it the entire set of gene expression products with *cis*-eQTL as well as all of the *cis*-eQTL genotypes as well. There is one tuning parameter, 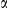, for the implementation ‘PCalg’, which represents the level of significance each test of conditional independence has to pass to correspond to removing an edge from the skeleton of the network. We used a conservative value of 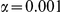, based on simulation results presented in Kalisch et al. [29]. For directed acyclic graphs, the PC-algorithm will also use the *cis*-eQTL to orient each of the edges in the network correctly and uniquely. For directed cyclic graphs, the PC-algorithm will try to orient the edges to form a directed acyclic graph, but often will fail, and draw a random DAG instead. We also apply the PC-algorithm to directed cyclic network recovery by having it identify both the skeleton with perturbations, and then have it attempt to orient as many edges as possible, given that every regulatory relationship should be orientable with the PC-algorithm when there are sufficient, unique perturbations. While in some cases this will fail, especially as the sample size grows and it becomes more sensitive to variations away from the assumption of no cycles, in practice it is able to orient many edges correctly in a directed cyclic graph.

**QDG algorithm**. The default settings were used for the QDG algorithm, as provided by the authors [11]: 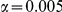 for the PC-algorithm skeleton reconstruction step, the skeleton reconstruction method based on the PC-algorithm, and the number of random restarts of iterative testing of different global edge orientations was set to ten. The QDG algorithm uses either the PC-algorithm or UDG algorithm [53] to generate a skeleton among phenotypes [12]. Then, the QDG algorithm orients edges between phenotypes based on a LOD score computed by leveraging each phenotype's known QTL. To find a globally optimal orientation of edges, an iterative search over orientations is performed to find all possibly cyclic networks which fit the data well [11]. We tried both methods in the QDG algorithm to generate the skeleton, and did not see a significant difference in performance for our simulations (results not shown).

**QTLnet algorithm**. The default settings were used for the QTLnet algorithm, as provided by the authors [16]: we ran it for 20,000 iterations, sampling every 20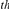 iteration after a burn-in of 2,000 iterations. The QTLnet algorithm uses a fully Bayesian Markov chain Monte Carlo approach to solve the problem of joint phenotype genotype network inference, constraining the proposed graph transitions to directed acyclic graphs [16]. In our analyses, we use the Bayesian model averaged output of the QTLnet algorithm, and include an edge only if its posterior probability of inclusion is greater than 0.50.

**NEO algorithm**. We used the default settings for the NEO algorithm, based on the code available from the author's website: http://www.genetics.ucla.edu/labs/horvath/aten/NEO/ [18]. The NEO algorithm uses multiple QTL to orient edges between an arbitrary pair of phenotypes based on different structural equation model based statistics [18], but has no mechanism to remove edges among phenotypes by conditioning on other phenotypes, and will therefore often have high false-discovery rate for recovery of the network generating the data among phenotypes. This was another justification, aside from the scaling of the algorithm, for why we did not include it in our broader comparison of alternative methods.
